# Characterization of CD44 intracellular domain interaction with RUNX2 in PC3 human prostate cancer cells

**DOI:** 10.1186/s12964-019-0395-6

**Published:** 2019-07-22

**Authors:** Linda T. Senbanjo, Hanan AlJohani, Sunipa Majumdar, Meenakshi A. Chellaiah

**Affiliations:** Department of Oncology and Diagnostic Sciences, University of Maryland Dental School, 650 W Baltimore St., 7th floor (south), Rm7207, Baltimore, MD 21201 USA

**Keywords:** Prostate cancer, Metastasis, CD44, RUNX2, CD44-ICD, MMP-9, OPN, Migration, Tumorigenesis

## Abstract

**Background:**

Expression of CD44 receptor is associated with the onset of several tumors. The intracellular domain of CD44 (CD44-ICD) has been implicated as a co-transcription factor for RUNX2 in the regulation of expression of MMP-9 in breast carcinoma cells. Previous studies from our laboratory demonstrated the role of CD44 in migration and invasion of PC3 prostate cells through activation of MMP-9. CD44 signaling regulates the phosphorylation and hence the localization of RUNX2 in the nucleus. The role of CD44-ICD has not been studied in prostate cancer cells. This study aimed to explore the role of CD44-ICD and RUNX2 in the regulation of expression of metastasis-related genes.

**Methods:**

PC3 and PC3 cells overexpressing RUNX2 protein were analyzed for RUNX2/CD44-ICD interaction by immunoprecipitation, immunoblotting, and Immunofluorescence analyses. Wound healing and tumorsphere formation analyses were also done in these cells. The real-time PCR analysis was used to detect the expression levels of different genes.

**Results:**

Expression of CD44 and RUNX2 was observed only in PC3 cells (androgen receptor positive) and not in LNCaP or PCa2b cells (androgen receptor negative). Therefore, CD44-ICD fragment (~ 15-16 kDa) was observed in PC3 cells. Moreover, localization of CD44-ICD was more in the nucleus than in the cytoplasm of PC3 cells. Inhibition of cleavage of CD44 with a γ-secretase inhibitor, DAPT reduced the formation of CD44-ICD; however, accumulation of CD44–external truncation fragments (~ 20 and ~ 25 kDa) was detected. RUNX2 and CD44-ICD interact in the nucleus of PC3 cells, and this interaction was more in PC3 cells transfected with RUNX2 cDNA. Overexpression of RUNX2 augments the expression of metastasis-related genes (e.g., MMP-9 and osteopontin) which resulted in increased migration and tumorsphere formation.

**Conclusions:**

We have shown here a strong functional relationship between CD44-ICD and RUNX2 in PC3 cells. RUNX2 forms a complex with CD44-ICD as a co-transcriptional factor, and this complex formation not only activates the expression of metastasis-related genes but also contributes to migration and tumorsphere formation. Therefore, RUNX2 and CD44-ICD are potential targets for anti-cancer therapy, and attenuation of their interaction may validate the regulatory effects of these proteins on cancer migration and progression.

## Background

Prostate cancer (PCa) is the second leading cause of death in men and the leading cause of non-skin cancer to affect men. It is also most commonly diagnosed in older men over the age of 65 [1–3]. PCa is characterized by extensive metastases leading to secondary lesions in the bone, lung, liver, brain, and adrenal [4–7]. Metastasis to secondary sites is often hard to treat partially due to the inadvertent failure of conventional androgen deprivation therapy (ADT) treatment [8, 9]. Specifically, ADT, as a treatment for metastatic PCa, demonstrated bone metastasis as well as osteopenia or osteoporosis [9, 10].

CD44, a cell surface receptor for hyaluronic acid (HA), osteopontin (OPN) and many other ligands has been shown to play a key role in prostate cancer (PCa) metastasis, migration, and invasion [6, 11, 12]. Interaction of CD44 with ligand(s) at the extracellular domain is responsible for controlling cellular signaling [13]. Expression of CD44 (standard or variant isoforms), is considered a prognostic marker for the progression of PCa [14]. However, the underlying molecular mechanisms by which CD44 regulates PCa progression, invasion, and metastasis still need further elucidation. In several types of cancers, including prostate cancer, CD44 is also a known marker of cancer stem cells (CSCs) or cancer-initiating cells [14, 15]. Cells that are positive for CD44 are capable of enhancing metastasis. These cancer stem cells have also been speculated to be representative of the subset of tumor cells that are responsible for metastatic disease and progression. CSCs have been shown to drive treatment failure and lead to the recurrence of the tumors [16, 17].

Sequential proteolytic cleavage of CD44 standard isoforms (CD44s) by MMPs and γ-secretase generates CD44-ICD long tail, which then translocates into the nucleus to regulate gene expression [12, 18, 19]. The sequential proteolytic cleavage is mediated by membrane-associated metalloproteases (MMPs) and subsequently by γ-secretase. The cleavage of the ectodomain fragment generates the amino-terminal fragment that can be released into culture supernatant as soluble CD44 and the membrane-bound carboxyl terminus fragment referred to as the CD44-EXT or extracellular truncation. The further proteolytic intramembranous cleavage generates the intracellular domain (CD44-ICD) fragment that then translocates into the nucleus to initiate transcription [6, 18, 20, 21]. As a result of CD44 cleavage, CD44 itself is one of the genes that can be transcribed [22] as well as MMP-9 [23] in breast cancer cells. CD44 is highly expressed in PC3 cells, which are androgen positive [3, 5, 24, 25]. However, CD44 expression was not observed in androgen receptor-positive prostate cancer cells derived from lymph node metastasis (LNCaP) or bone metastasis (PCa2b). CD44 expression was reduced in PC3 cells transfected with androgen receptors [7]. Androgen receptor modulates the expression of CD44 in prostate cancer cells. Studies by others have identified CD44 cleavage product (CD44-ICD) in complex with RUNX2 on the promoter of the MMP-9 gene in breast cancer cells [23]. The role of CD44-ICD has not been studied in prostate cancer cells. The present study aims to characterize whether CD44-ICD is formed in androgen negative PC3 cells and has a role as a co-transcriptional factor with RUNX2.

RUNX2, a transcription factor and master regulator of bone formation, is highly expressed in tumor cells that metastasize to bone [26–28]. Specifically, RUNX2 has been shown to regulate genes (e.g., MMP2 and MMP-9) that are involved in metastasis-related events of prostate and breast cancer cells [3, 26]. Though RUNX2 is highly expressed in metastatic breast and prostate cancer cells, its expression in the normal breast or prostate epithelial cells is negligible [27–29]. Dose-dependent knockdown of RUNX2 led to a decrease in MMP-9 expression but not MMP2 [3]. Our previous studies have shown that CD44 signaling in PCa cells regulates the phosphorylation of RUNX2 and knockdown of CD44 reduced RUNX2 phosphorylation [3]. However, CD44 interaction with RUNX2 has not been studied in PCa cells.

In this study, we aimed to identify the role of CD44-ICD and its interaction with RUNX2 in metastasis and tumorigenesis. We found that CD44 and RUNX2 were highly expressed in PC3 cells as compared to LNCaP or PCa2b cells. CD44 was cleaved to generate CD44-ICD which interacted with RUNX2 in the nucleus. CD44-ICD/RUNX2 interaction was not only more in PC3 cells overexpressing RUNX2 but also increased the migration and tumorsphere formation in vitro. Taken together, our observations suggest that CD44-ICD may be an important co-factor for RUNX2-mediated transcriptional regulation.

## Materials and methods

### Materials

Antibodies to CD44 (156-3C11), RUNX2 (D1L7F), SOX2 (D6D9), Ezrin (3145S) MMP-9 (D6O3H) and Nucleoporin (C39A3) were purchased from Cell Signaling Technology, Inc. (Danvers, MA). Androgen Receptor (sc-7305) and RUNX2 (sc-390351) antibodies were purchased from Santa Cruz Biotechnology, Inc. (Dallas, TX). CD44-ICD antibody (KAL-KO601) was purchased from Cosmo Bio (Tokyo, Japan). Antibody to GAPDH (G9545) and other chemicals were purchased from Sigma-Aldrich, Inc. (St.Louis, MO). HRP-conjugated Anti-Rabbit and anti-Mouse secondary antibodies were obtained from Kirkegaard & Perry Laboratories (Gaithersburg, MD) and Santa Cruz Biotechnology (Dallas, TX), respectively. Protein estimation reagent, molecular weight protein standards, and reagents for polyacrylamide gel electrophoresis were purchased from Bio-Rad (Hercules, CA). Polyvinylidene difluoride membrane was obtained from Millipore Corp. (Bedford, MA). ECL reagent was purchased from Pierce (Rockford, IL). The fluorochrome-conjugated secondary antibody Alexa Fluor 488 (4412) and ProLong Gold Antifade DAPI (8961) were obtained from Cell Signaling Technology, Inc. (Danvers, MA).

### Cells

Human prostatic carcinoma cell lines such as, PC3 (derived from Caucasian bone metastasis), PCa2b (derived from African American bone metastasis), LNCaP (derived from lymph node metastasis), PC3/AR+ (PC3 cells stably expressing AR cDNA [7]), PC3/RUNX2 (PC3 cells stably expressing RUNX2 cDNA) were used. Normal prostatic epithelial cells (HPR1) and normal human epithelial cells (RWPE1) were used as controls.

### Cell culture

PC3 and LNCaP cells were cultured in Roswell Park Memorial Institute (RPMI)-1640 medium containing 10% fetal bovine serum (FBS), as previously described [5, 24]. HPR1 and RWPE1 cells were cultured in keratinocyte medium supplemented with epidermal growth factor (2.5 mg/500 mL) and bovine pituitary extracts (25 mg/500 mL) (Gibco, Life Technologies, Bethesda, MD). PCa2b was cultured in BRFF-HPC1 (Athena ES, Baltimore, MD) medium containing only 10% FBS, slightly modified from previously described [30]. Heat-inactivated FBS (16000036; Gibco, Life Technologies) was used to culture PCa2b cells. All cell culture media were supplemented with 1% penicillin and streptomycin; cells were maintained at 37 °C in a humidified incubator with 5% CO2.

### RNA extraction and quantitative real-time PCR

Total RNA extraction from PC3, LNCaP, PCa2b, and PC3 cells overexpressing RUNX2 (PC3/RUNX2) and real-time RT-PCR analysis was performed as described [3, 7]. SYBER Universal Master Mix (Applied Biosystems, Foster City, CA) was used along with custom real-time PCR primers for CD44 (NM_000610.3) [7], RUNX2 [3], MMP-9 (NM_004994.2) [31], SOX2 [7], OCT4 [7], and GAPDH [3]. The forward (F) and reverse (R) primers used for the indicated genes are as follows:

**Table:** 

CD44	F: 5′-ACCGACAGCACAGACAGAATC-3′
	R: 5′-GTTTGCTCCACCTTCTTGACTC-3′ [7]
RUNX2	F: 5′-CGGCCCTCCCTGAACTCT-3′
	R: 5′-TGCCTGCCTGGGGTCTGTA-3′ [3]
MMP-9	F: 5′-CTGTCCAGACCAAGGGTACAGCCT-3′
	R: 5′-GAGGTATAGTGGGACACATAGTGG-3′ [31]
OPN	F: 5′-CCACAGTAGACACATATGATGG-3′
	R: 5′-CAGGGAGTTTCCATGAAGCCAC-3′ [32]
OCT4	F: 5′-TCGAGAACCGAGTGAGAGG-3′
	R: 5′-GAACCACACTCGGACCACA-3′ [7]
SOX2	F: 5′-AACCCCAAGATGCACAACTC-3′
	R: 5′-CGGGGCCGGTATTTATAATC-3′ [7]
GAPDH	F: 5′-TGCACCACCAACTGCTTAG-3′
	R: 5′-GATGCAGGGATGATGTTC-3′. [3]

### Lysate preparation and immunoblotting analysis

Cells were washed two times in cold 1X PBS and lysed in lysis buffer (62.5 mM Tris-HCl ph 7.5; 10% glycerol, and 2% SDS). Lysates were collected, then sonicated for 30 s and centrifuged for 5 min at 14, 000 rpm at room temperature. The supernatants were used for protein estimation and immunoblotting analysis, as previously described with the following modification [33]. The samples were heated at 70 °C for 15 min prior to loading on the gel.

### Cytoplasmic and nuclear protein fraction preparation

Cytoplasmic and nuclear protein fractions were isolated from PCa cell lines using nuclear extraction kit (ab113474; Abcam Biotechnology, Cambridge, United Kingdom) according to the manufacturer’s recommendations.

### Immunoprecipitation analysis

Equal amounts of protein lysates (50–150 μg) were used for immunoprecipitation analysis. Immunoprecipitation analysis was done as described previously [34, 35].

### Overexpression of RUNX2 in PC3 cells

PC3 cells were grown in 6-well plates overnight at 37 °C and allowed to reach ~ 80% confluency. HA-RUNX2 construct was transfected using Lipofectamine 2000 (ThermoFisher Scientific). After 24 h of transfection, cells were kept in RPMI media with 10%FBS. After 24 h, cell lysates were collected and subjected to SDS-PAGE. Immunoblotting analysis was done to confirm the overexpression of RUNX2. The stable selection was done for three weeks with 100 μg G418 Sulfate (30–234-CR) Corning Inc. (Corning, NY).

### Immunostaining analysis

Immunostaining and image analyses of cells were done as described [7]. Antibodies were used in the following dilutions in antibody dilution buffer (1× PBS/1% bovine serum albumin/0.3% Triton X-100): RUNX2 (1:100 dilution), CD44 (1:1000 dilution) and the fluorochrome-conjugated secondary antibody (1:500 dilution). Stained cells were imaged on Zeiss LSM 510 META Confocal Laser Scanning Microscopes (Zeiss, Germany).

### Wound closure and tumorsphere formation assays

Wound closure and tumorsphere formation assays were done as described [7]. Mitomycin C (10 μg/ ml) was added to the medium to inhibit proliferation of cells during migration in the wound closure assay [5, 7]. Wound closure was monitored by the migration of cells for 24 h; pictures were taken at 0 h, 8 h and 24 h time points with a digital SPOT camera attached to an inverted Nikon phase contrast microscope. Cells were incubated for seven days to induce tumorsphere formation as described [7, 36, 37]. Tumorspheres were imaged using a Cytation-3 Imaging Reader from Biotek.

### Statistical analysis

All values presented as mean ± SEM. A value of *p* < 0.05 was considered significant. Two-tailed Student’s t-test determined statistical significance. All of the data were analyzed with GraphPad Prism (GraphPad Software, Inc., La Jolla, CA).

## Results

### CD44 is expressed both at the mRNA and protein levels in PC3 cells

**Fig. 1:**
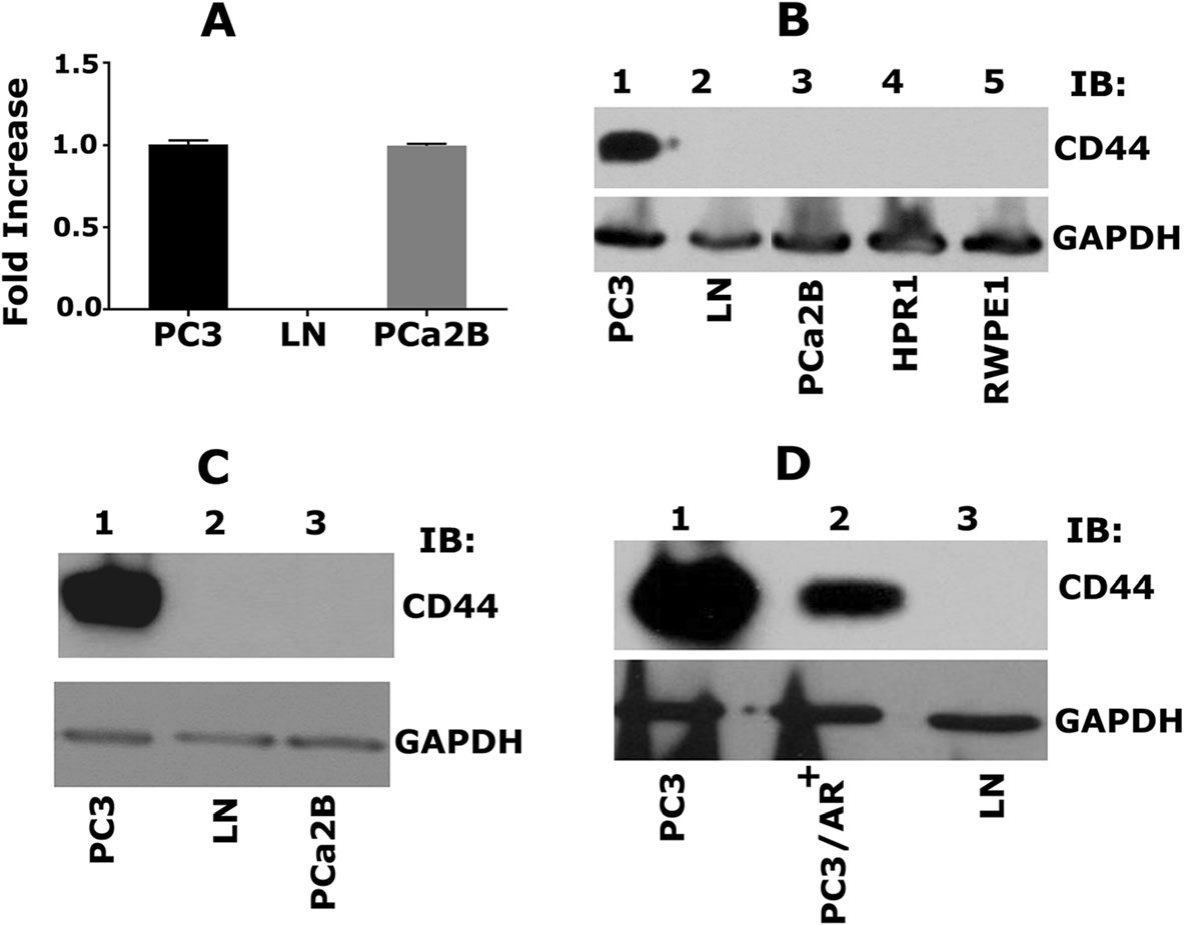
Characterizing the expression of CD44 in PCa cell lines. **a**. Real-time PCR analysis of CD44 expression in PC3 (lane 1), LNCaP (lane 2), and PCa2b (lane 3) cells. **b**. Immunoblotting (IB) analysis with an antibody to CD44 (top panel) and GAPDH (bottom panel). Equal amounts of protein lysates (40 μg) made from PC3 (lane 1), LNCaP (lane 2), PCa2b (lane 3), control HPR1 and RWPE1 (lane 4 & 5) cells were immunoblotted with CD44 antibody to detect total cellular levels. **c**. Equal amounts of PC3, LNCaP, PCa2b cells were immunoblotted with CD44 antibody. **d**. Equal amounts of PC3 (lane 1), PC3/AR+ (lane 2), and LNCaP (lane 3) were immunoblotted with CD44 antibody to detect total cellular levels. GAPDH was used as a loading control for real-time PCR and immunoblotting analysis (**a**-**c**). The results represent one of three separate experiments performed with the same results

Previous studies in our lab have identified varying expression levels of CD44 in PCa cell lines derived from different metastasis [3]. We sought to validate the expression levels of CD44 in indicated cell lines of interest before proceeding our studies. We used real-time PCR and immunoblotting analyses (Fig. 1). As shown previously, CD44 mRNA and protein levels were high in PC3 cells (Fig. 1a-c) but negligible or not observed in LNCaP cells (Fig. 1 a-c). Also, CD44 protein was not observed in control normal prostatic epithelial cell lines such as HPR1 and RWPE1 (Fig. 1b). Interestingly, androgen receptor positive PCa2b cells expressed CD44 at mRNA levels (Fig. 1a) but not at protein levels (Fig. 1b and c, lane 3). We then used PC3 cells stably expressing androgen receptor cDNA (PC3/AR+). PC3/AR+ and LNCaP cells demonstrated significantly decreased levels of CD44 (Fig. 1d, lane 2 and 3). Our results suggest that AR expression in PCa cell lines has differential effects on the expression of CD44.

### RUNX2 is expressed both at the mRNA and protein levels in PC3 cells

**Fig. 2:**
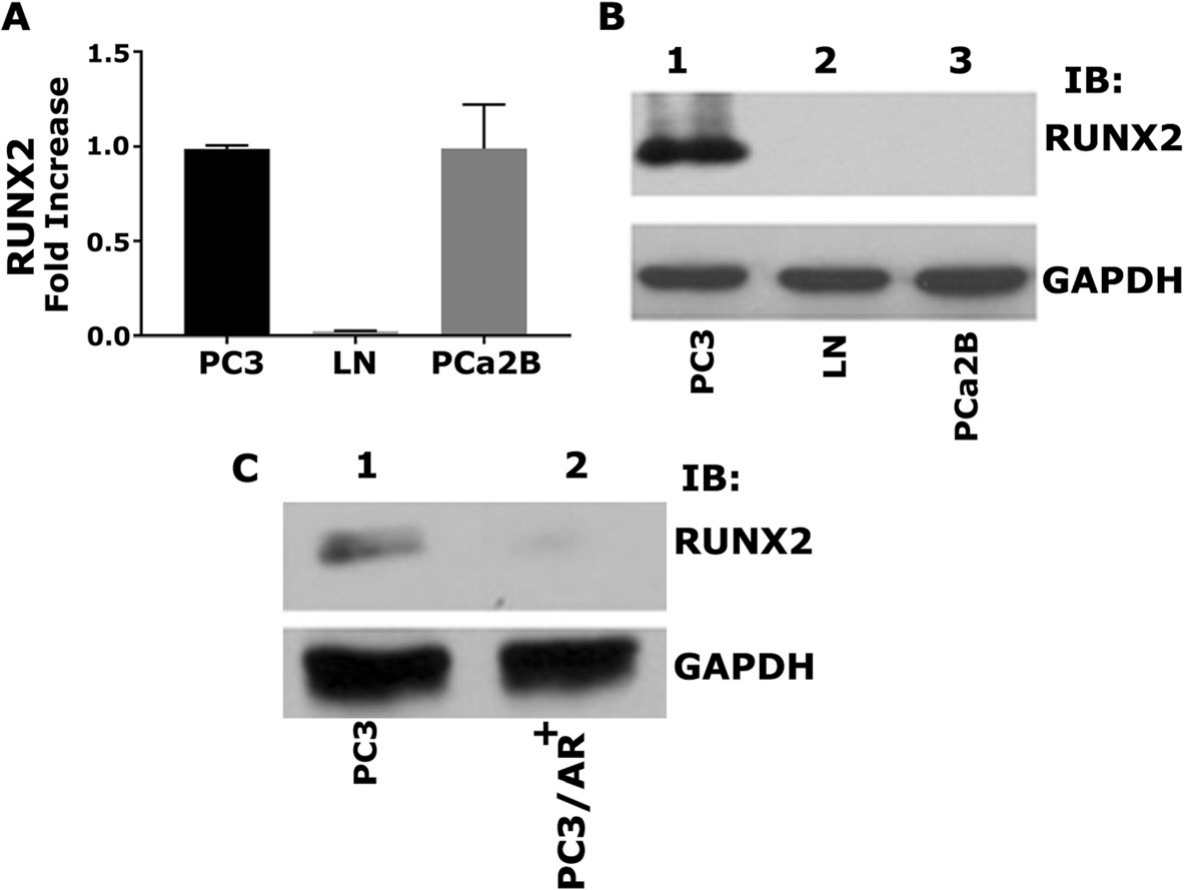
Characterizing the expression of RUNX2 in PCa cell lines. **a**. The real-time PCR analysis of RUNX2 expression in PC3 (lane 1), LNCaP (lane 2), and PCa2b (lane 3) cells. **b**. Immunoblotting analysis with an antibody to Runx2 (top panel) and GAPDH (bottom panel). Equal amounts of protein lysates (40 μg) made from PC3 (lane 1), LNCaP (lane 2), PCa2b (lane 3). **c**. Equal amounts of PC3 and PC3/AR+ were immunoblotted with RUNX2 antibody. GAPDH was used as a loading control for real-time PCR and immunoblotting analysis (**a**-**c**). The results represent one of three separate experiments performed with the same results

We have previously shown that knockdown of CD44 in PC3 cells (CD44−/−) decreased RUNX2 at mRNA and protein levels as well as RUNX2-mediated transcriptional regulation [3]. Therefore, we wanted to determine the expression levels of RUNX2 in PCa cells of interest. RUNX2 was highly expressed both at mRNA (Fig. 2a) and protein (Fig. 2b, lane 1) levels in PC3 cells, but not in LNCaP cells (Fig. 2a and b; lane 2). Again surprisingly, RUNX2 level was elevated in PCa2b cells at mRNA levels but not expressed at the protein level (Fig. 2a and b, lane 3). RUNX2 expression was very low or not observed in PC3/AR+ cells (Fig. 2c; lane 2). These results suggest the possibility that RUNX2 expression is dependent on CD44 mediated signaling. The present study aims to characterize the interaction of CD44-ICD with RUNX2. Our initial characterizations (Figs. 1 and 2) demonstrated that PC3 cells are the only cells that express both CD44 and RUNX2 at mRNA and protein levels. Therefore, studies described below used PC3 cells.

### CD44 is sequentially cleaved by MMP2 and γ-secretase to generate the CD44-ICD fragment

Sequential proteolytic cleavage of CD44 by MMPs and γ-secretase was shown to generate CD44-ICD, which then translocates into the nucleus to regulate gene expression [12, 18]. As a requirement for studies on the interaction of CD44-ICD with RUNX2, we first tested for the antibody which recognizes correct CD44-ICD fragment and second the predominant localization of CD44-ICD in PC3 cells by immunoblotting analyses.

**Fig. 3:**
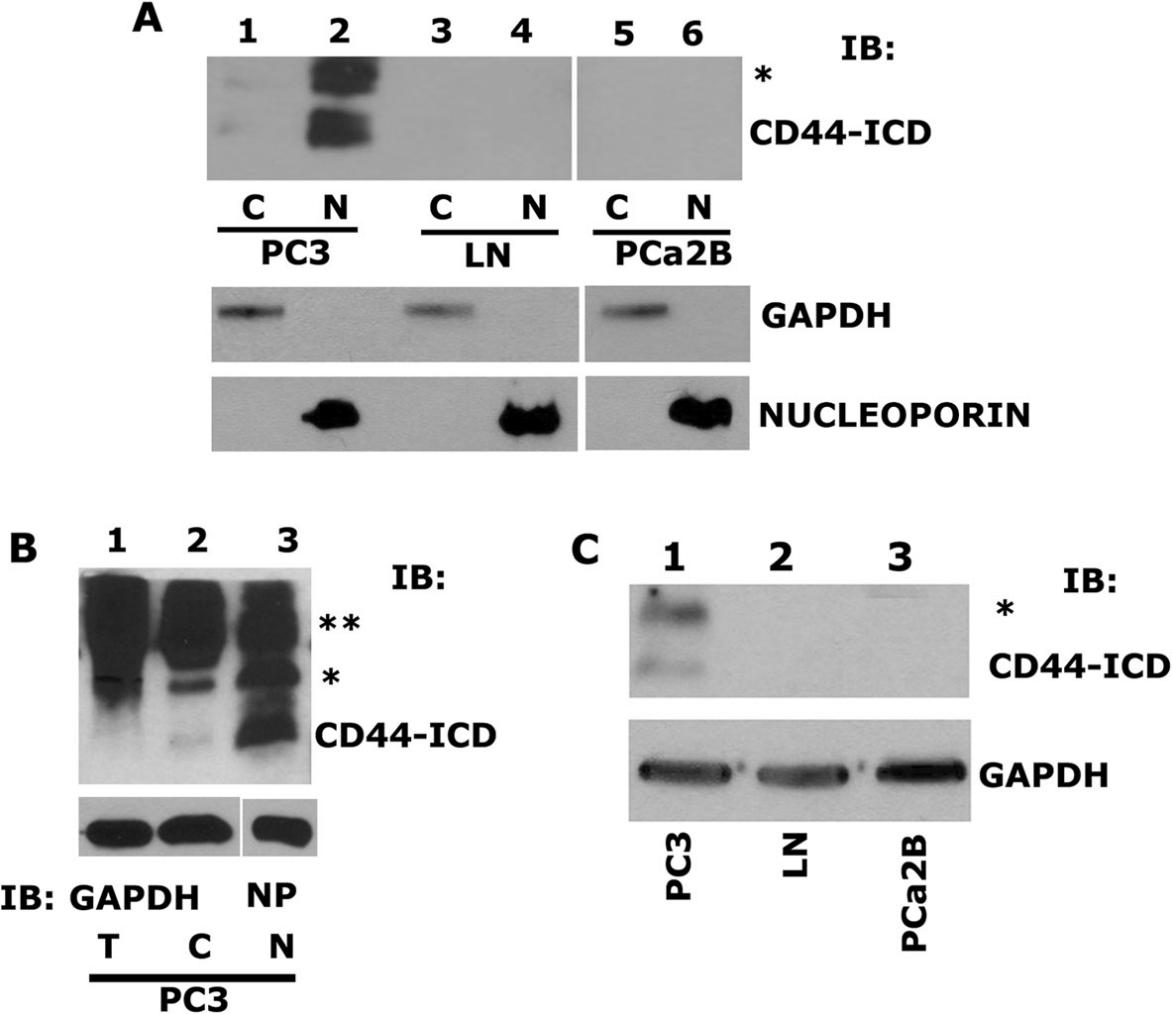
Characterizing the expression of CD44-ICD in PCa cell lines. **a**. Immunoblotting analysis with an antibody to CD44-ICD (top panel) and GAPDH (bottom panel). Equal amounts of protein lysates (40 μg) made from PC3 (C) – cytoplasmic fraction (lane 1), PC3 (N) – nuclear fraction (lane 2), LNCaP (C) – cytoplasmic fraction (lane 3), LNCaP (N) – nuclear fraction (lane 4), PCa2b (C) – cytoplasmic fraction (lane 5), PCa2b (N) – nuclear fraction (lane 6) were immunoblotted with CD44-ICD antibody to detect cytoplasmic and nuclear levels. **b**. IB analysis of total (T), cytoplasmic (C) and nuclear (N) lysates (20μg) from PC3 cells with an antibody to CD44-ICD (~ 16.5 kDa). **c**. IB analysis of total cellular lysates (40μg) from PC3 (lane 1), LNCaP (lane 2) and PCa2b (lane 3) cells were immunoblotted for antibody to CD44-ICD. GAPDH was used as a loading control for immunoblotting analysis (A, lane 1, 3 & 5 and B, lane 1 & 2 and C). Nucleoporin (NP) was used as a loading control for nuclear (N) lysates (A, lane 2, 4 & 6, and B, lane 3). The results represent one of three separate experiments performed with the same results. Two asterisks (**) represents the 25 kDa CD44 extracellular truncation (CD44-EXT) while one asterisk (*) represents the 20 kDa CD44 extracellular truncation (CD44-EXT)

We showed here that CD44-ICD antibody (Cosmo Bio) recognized CD44 protein fragments of ~ 20 kDa (indicated by an asterisk) and ~ 16 kDa (CD44-ICD) (Fig. 3a). Also, CD44 fragments were more in the nuclear fraction (Fig. 3a, lane 2) than in cytoplasmic fraction of PC3 cells (lane 1). As anticipated, LNCaP and PCa2b cells were negative for CD44-ICD fragments (lanes 3–6). To corroborate the nuclear localization of CD44-ICD, we used total cellular lysate as well as cytoplasmic and nuclear fractions of PC3 cells. Immunoblotting analysis with a CD44-ICD antibody demonstrated a population of CD44 fragments which were more in the nuclear fraction (Fig. 3b, lane 3) than in the cytoplasmic fraction or total cellular lysate (lanes 1 and 2) of PC3 cells. These observations again confirmed the predominant localization of CD44-ICD in the nucleus after cleavage (Fig. 3b). Immunoblotting with a GAPDH and nucleoporin demonstrated the purity of the cytoplasmic and nuclear fractions. These were also used as loading controls (Fig. 3a and b).

To further identify the detectable levels of CD44-ICD fragment in the total cellular fragment, we used more total cellular lysate proteins. CD44 protein fragments of ~ 20 kDa (indicated by an asterisk) and ~ 16 kDa (CD44-ICD) were observed, but the levels were considerably lower than the level observed in the nuclear fraction (Fig. 3b, lane 3). Consistently, CD44-ICD fragment was observed only in PC3 cells (Fig. 3c, lane 1). CD44-ICD has the ability to localize to the nucleus, which is one of the basic principles for a protein to be a regulator of transcription.

### The cleavage and formation of CD44-ICD regulates RUNX2 expression

**Fig. 4:**
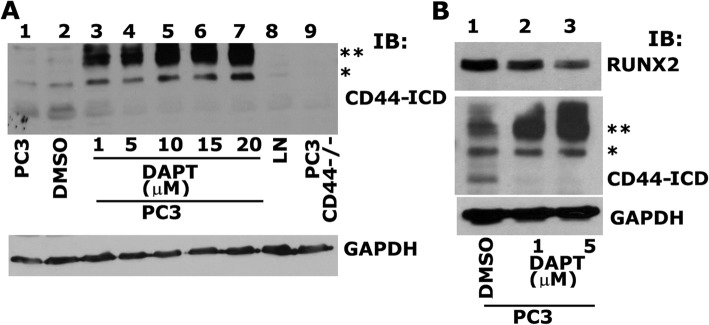
Analysis of the effect of γ-Secretase inhibitor, DAPT on PC3 cells. **a**. Equal amounts of total lysates (40μg) were immunoblotted for CD44-ICD. IB analysis of PC3 cells untreated, PC3 cells treated with DMSO control, PC3 cells treated with increasing concentrations (1 μM - 20 μM) γ-secretase inhibitor (DAPT) lanes 3–7, LNCaP cells, and PC3 cells knockdown of CD44 was immunoblotted with an antibody to CD44-ICD (top panel) and GAPDH (bottom panel). **b**. PC3 cells treated with DMSO control (lane 1), 1 μM of DAPT (lane 2) and 5 μM of DAPT (lane 3) was immunoblotted with an antibody to RUNX2 (top panel), CD44-ICD (middle panel) and GAPDH (bottom panel). Two asterisks (**) represents the 25 kDa CD44 extracellular truncation (CD44-EXT) while one asterisk (*) represents the 20 kDa CD44 extracellular truncation (CD44-EXT)

We have previously shown the relationship of CD44 signaling to RUNX2 expression. CD44−/− cells demonstrated reduced expression of RUNX2 at the mRNA and protein levels [3]. Therefore, we proceeded to determine whether CD44-ICD interacts with RUNX2 and whether abrogation of this interaction will reduce RUNX2 levels. Here, we blocked the cleavage of CD44 using an inhibitor to γ-secretase (DAPT). Dose-dependent effects of DAPT on CD44-ICD formation is shown (Fig. 4a). Immunoblotting analysis was done with an antibody to CD44-ICD in PC3 cells (Fig. 4a, lane 1), PC3 cells treated with DMSO (lane 2), PC3 cells treated with indicated concentrations of DAPT (lanes 3–7), LNCaP cells (lane 8) and CD44−/− cells (lane 9). A dose-dependent decrease in the cleavage and formation of CD44-ICD fragment was observed at 1 μM, and 5 μM DAPT (Fig. 4, lanes 3 and 4) and this decrease was stabilized at 10, 15 and 20 μM DAPT (lanes 5–7). Formation of two extracellular truncation fragments (CD44-EXT) with molecular weight (MW) ~ 25 kDa (**) and ~ 20 kDa (*) was observed in PC3 cells treated with DAPT (lanes 3–7). Formation of these fragments was very minimal in untreated (lane 1) or DMSO-treated (lane 2) PC3 cells. CD44-ICD fragment was observed in these cells (lanes 1 and 2).

To determine the effect of DAPT on the expression of RUNX2 expression, we chose 1 μM and 5 μM concentration of DAPT (Fig. 4b, lanes 2 and 3). A dose-dependent decrease in RUNX2 expression was observed in DAPT treated PC3 cells (Fig. 4b, lanes 2 and 3) as compared to DMSO-treated PC3 cells (lane 1). A decrease in the levels of RUNX2 in DAPT-treated cells (Fig. 4b) and CD44−/− cells [1] is interesting. Taken together, CD44 cleavage by γ-secretase appeared to have a role in the formation of CD44-ICD; subsequently, it may have a role in RUNX2 expression.

### RUNX2 interacts with CD44-ICD in the nucleus of PC3 cells

**Fig. 5:**
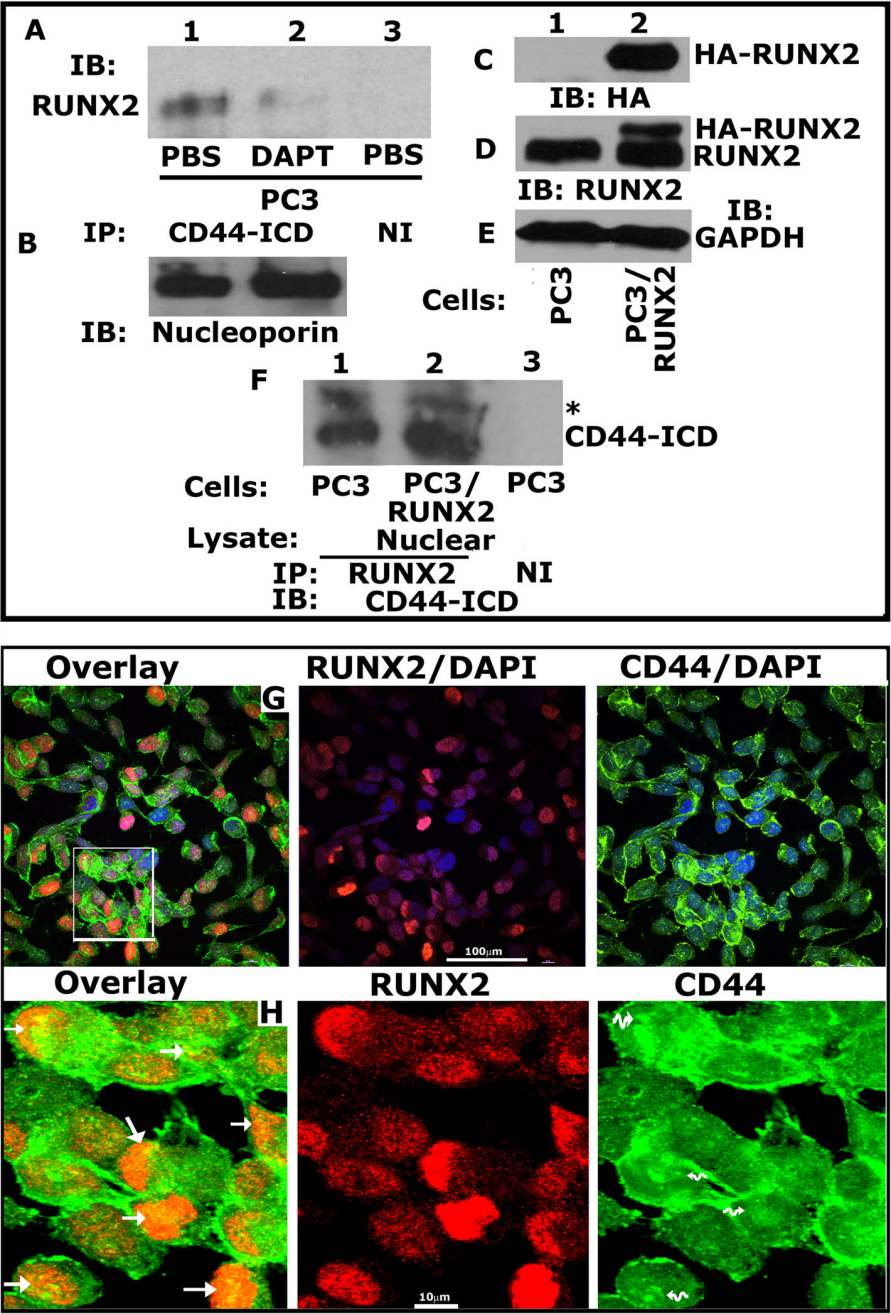
Analysis of the interaction between CD44-ICD and RUNX2. **a**. Equal amounts of nuclear lysates of PC3/PBS (lane 1 and 3) and PC3/DAPT (lane 2) were immunoprecipitated with CD44-ICD antibody (lane 1 and 2) and subjected to immunoblotting (IB) analyses with an antibody to RUNX2 (lane 1–3). **b**. Immunoblotting analysis of the nuclear lysates from PC3 cells treated with PBS or DAPT (panel B) with a nucleoporin antibody demonstrates an equal amount of nuclear proteins were used for immunoprecipitation analysis shown in Fig. 5a. **c-e**. PC3 cells (lane 1) and PC3/RUNX2 overexpressing cells (lane 2) were immunoblotted with an HA- (C), RUNX2 and GAPDH (E) antibody. **f**. Equal amounts of PC3 (lane 1 and 3) and PC3/RUNX2 (lane 2) cells were immunoprecipitated with an antibody to RUNX2 and subjected to IB analyses with a CD44-ICD antibody. One asterisk (*) represents the 20 kDa CD44 extracellular truncation fragment (CD44-EXT). **g-h**: Analysis of the localization of CD44 (green), RUNX2 (red), and DAPI (a nuclear counterstain; blue) in PC3 cells. **g**. Confocal microscopy shows overlay stainings for CD44/DAPI/RUNX2 (green/blue/red), RUNX2/DAPI (red/blue), and CD44/DAPI (green/blue) in PC3 cells. Scale bar-100 μm. **h**. The rectangle in panel G defines the area of the image which is magnified in panel H. Arrows point to regions of colocalization (yellow) of CD44 fragment “CD44-ICD” (green) and RUNX2 (red); wavy arrows (H; CD44 panel) point to areas where CD44-ICD is localized in the nucleus and colocalized with RUNX2 in the overlay panel. Scale bar-10 μm. These results represent one of the three experiments performed with similar results

To determine if RUNX2 and CD44-ICD interact in the nucleus of PC3 cells, we performed immunoprecipitation and confocal analyses (Fig. 5). Immunoprecipitation analysis was performed in nuclear fractions of PC3 cells treated with PBS (Fig. 5a, lane 1) or DAPT (lane 2) with antibodies to CD44-ICD (Fig. 5a, lane 1 and 2). Immunoprecipitates made with a species-specific non-immune (NI) serum was used as a control for immunoprecipitation (lane 3). Immunoblotting with a RUNX2 antibody demonstrated co-precipitation of RUNX2 (~ 56 kDa) in PBS-treated cells (Fig. 5a, lane 1). Coprecipitation is significantly lower in DAPT-treated cells (lane 2). RUNX2 protein was not observed in the immunoprecipitates made with a NI serum (Fig. 5a, lane 3). We used nucleoporin immunoblot as a loading control for the IP samples (Fig. 5b). Further indicating the role CD44-ICD may have in the expression of RUNX2.

To further elucidate CD44-ICD/RUNX2 interaction in the nucleus, we generated stable PC3 cells expressing HA-tagged RUNX2 cDNA (PC3/RUNX2). Using total cellular lysates, we confirmed the expression of HA-tagged RUNX2 by immunoblotting with an antibody to HA (Fig. 5c) and RUNX2 (Fig. 5d). Immunoblotting with an HA-antibody showed expression of HA-tagged RUNX2 only in PC3/RUNX2 cells (Fig. 5c, lane 2) and not in control PC3 cells (lane 1). Immunoblotting with a RUNX2 antibody substantiated this observation. It recognized both HA-tagged (~ 59-60 kDa) and endogenous RUNX2 (~ 56 kDa; Figure D, Lane 2). Only endogenous RUNX2 was observed in control PC3 cells (D, lane 1). The MW of HA-tag is ~ 1102.15 Da (~ 1.1 kDa). A 3 kDa shift in HA-tagged RUNX2 (~ 59-60 kDa) may be due to reduced mobility of the fusion proteins in SDS-PAGE by HA-tag as shown by others [38]. Subsequently, immunoprecipitates made from PC3 (lane 1) and PC3/RUNX2 cells (lane 2) were used for immunoblotting with a CD44-ICD antibody (Fig. 5f). Here we showed co-precipitation of CD44-ICD (16 kDa) and 20 kDa (indicated by an asterisk) fragments of CD44 with RUNX2. While the levels of 20 kDa fragment remain the same in both PC3 and PC3/RUNX2 cells, its association with RUNX2 is considerably lower than CD44-ICD fragment (Fig. 5f, lanes 1 and 2). An increase in the expression of RUNX2 corresponded with an increase in the co-precipitation of CD44-ICD in PC3/RUNX2 cells (lane 2) as compared with PC3 cells (lane 1). Neither RUNX2 (Fig. 5a, lane 3) nor CD44-ICD (Fig. 5f, lane 3) was observed in the immunoprecipitates made with a species-specific non-immune serum (NI).

RUNX2/CD44-ICD interaction was also confirmed in PC3 cells by immunostaining analysis with an antibody to CD44 and RUNX2 (Fig. 5g-h). DAPI staining (blue) was used to evaluate the nuclear localization of CD44 and RUNX2. Colocalization of CD44/RUNX2/DAPI is shown in the overlay (yellow; Fig. 5g). High magnification regions are shown in the bottom panel (Fig. 5h) and is indicated by a corresponding rectangular field in the top panel (Fig. 5g). Although diffused cytoplasmic staining of CD44 was observed in PC3 cells (Fig. 5h, right panel), intense staining of CD44 in the nucleus (indicated by wavy arrows in the right panel) represents the nuclear localization of CD44-ICD fragment. Interestingly, this is colocalized with RUNX2 in the nucleus (yellow; Fig. 5h, left panel; indicated by arrows). No colocalization of cytoplasmic CD44 with RUNX2 again confirmed the specificity of interaction of fragments of CD44 with RUNX2. Based on the above observations, we propose that CD44-ICD/RUNX2 interaction is well-maintained in the nucleus of PC3 cells.

### RUNX2 overexpression upregulates the expression of metastasis-related genes in PC3 cells

**Fig. 6:**
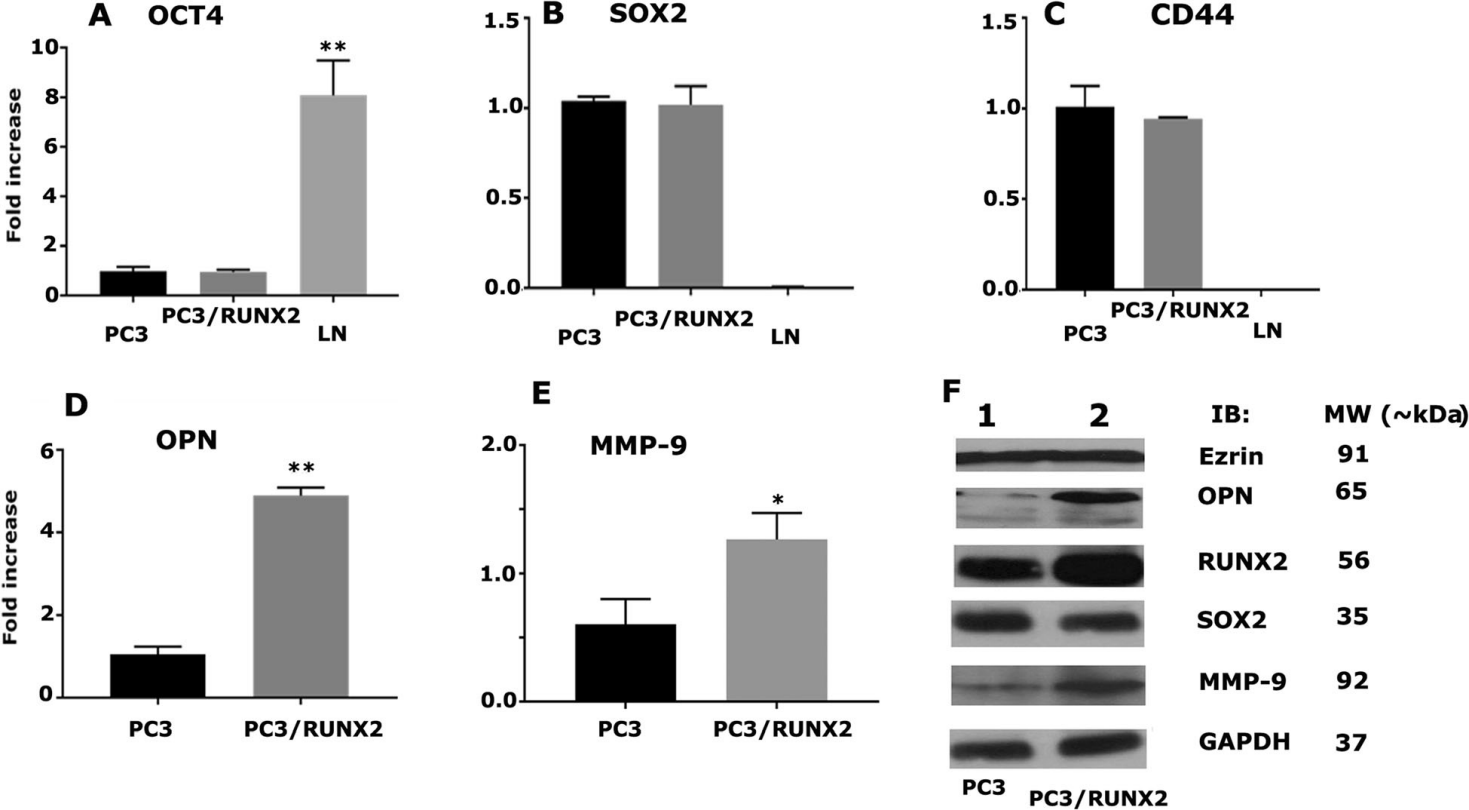
The effect of RUNX2 overexpression on the expression of metastasis-related genes. **a-e**. The real-time PCR analysis of OCT4, SOX2, CD44, OPN, and MMP9 expression in PC3, PC3 cells transfected with RUNX2, and LNCaP cells. **f**. An equal amount of protein lysates (30 μg) made from PC3 (lane 1) and PC3 cells transfected with RUNX2 (lane 2) were immunoblotted with Ezrin, OPN, RUNX2, SOX2, MMP9 antibodies to detect total cellular levels of the respective proteins. GAPDH was used as a loading control for real-time PCR and IB analysis (A-G). The results represent one of three separate experiments performed with the same results

Here, our goal was to determine the effect of RUNX2 overexpression on the expression of metastasis-related genes (Fig. 6). RUNX2 overexpression did not have any significant effect on the mRNA levels of OCT4 (Fig. 6a), SOX2 (B), and CD44 (C) genes. However, an increase in osteopontin (OPN; Fig. 6d) and MMP-9 (Fig. 6e) was observed at mRNA levels in PC3/RUNX2 cells, compared to PC3 cells. Likewise, at protein levels, we did not observe a significant increase in the expression of ezrin or SOX2 (Fig. 6g); but we observed a marked increase in the expression of OPN and MMP-9 in PC3/RUNX2 cells, compared to PC3 cells. These results are consistent with previous studies by others that RUNX2 overexpression increases the expression of genes that are crucial for metastasis of breast cancer cells [26, 39].

### RUNX2 overexpression promotes wound healing and tumorsphere formation in PC3 cells

**Fig. 7:**
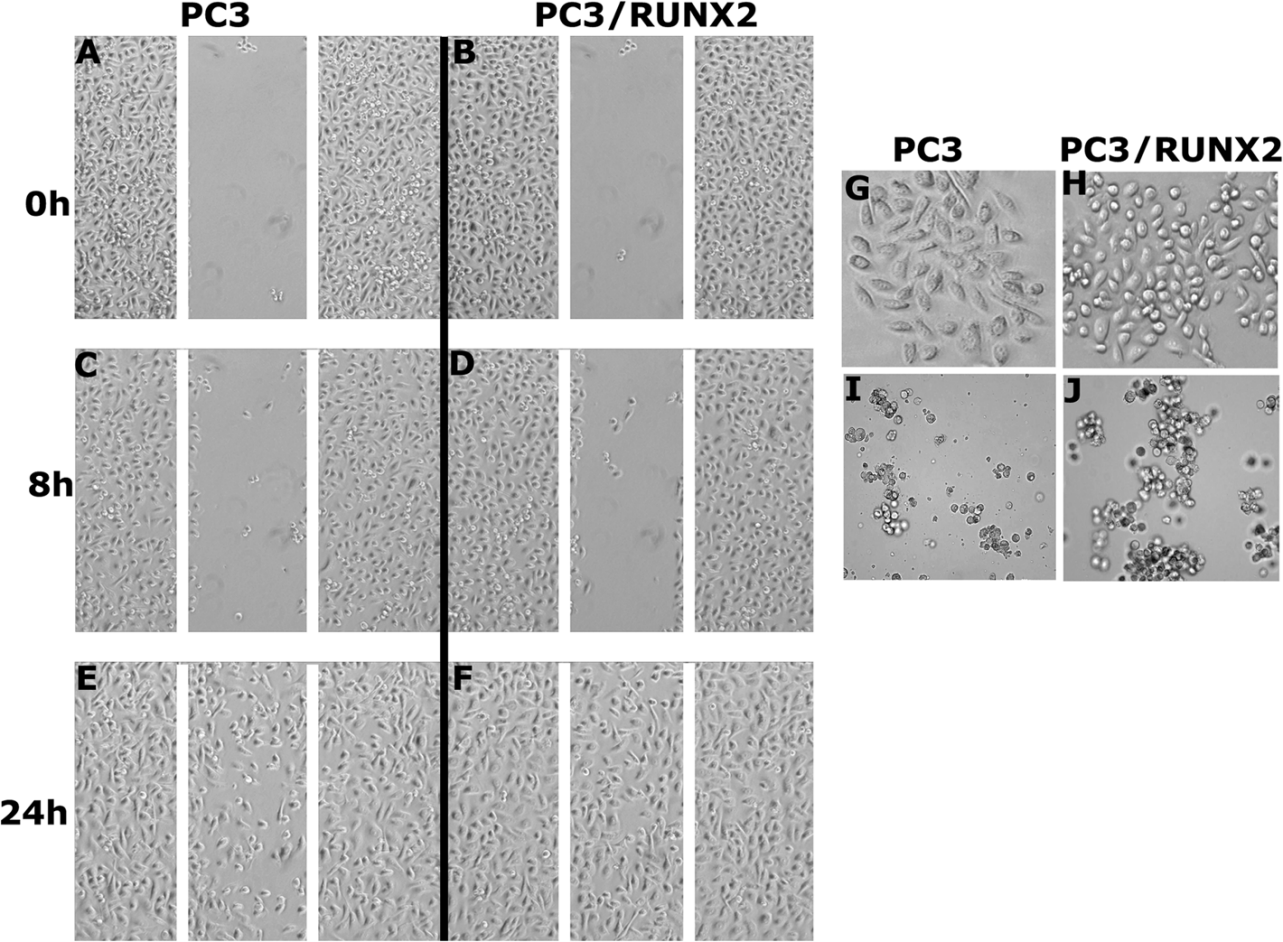
Analysis of the effect of RUNX2 overexpression on migration, cell morphology, and tumorsphere formation in PC3 cells. **a-f**. PC3 and PC3/RUNX2 cells were subjected to wound closure assay. Phase contrast micrographs show migration at 0, 8, and 24 h. **g-h**. Phase contrast micrographs show the morphology of PC3 and PC3/RUNX2 cells at × 100 magnification. **i-j**. Cell imaging in the multimode microscope (cytation3) shows tumorsphere formation in PC3 and PC3/RUNX2 cells. Scale bar: 200 μm. The results are representative of three independent experiments

To further define and highlight the impact of RUNX2 overexpression on metastasis, we performed wound healing and tumorsphere formation assays in vitro in PC3 and PC3/RUNX2 cells. Wound closure was monitored for 8 and 24 h (Fig. 7). PC3/RUNX2 cells displayed greater migration and wound closure capabilities (Fig. 7f) as compared to PC3 cells (Fig. 7e) at 24 h. While PC3 cells were spindle-shaped (Fig. 7g), PC3/RUNX2 cells demonstrated rounded morphology. Also, some of them displayed polygonal well-spread morphology (Fig. 7h).

Then we sought to determine if RUNX2 overexpression influences tumorsphere formation in vitro in PC3 cells. PC3/RUNX2 cells had a greater capability of proliferating and forming colonies as compared to PC3 cells. The round shaped morphology in PC3/RUNX2 cells could support aggregate and sphere formation.

## Discussion

The mechanism of CD44 action has been studied extensively in cancer cells. It is a key regulator of metastasis through its interaction with its several ligands [5, 11, 40, 41]. Additionally, recent studies have been directed towards CD44-ICD as the main regulator of metastasis in cancer cells through its interaction with transcription factors that regulate expression of genes involved in metastasis [18, 23, 42]. However, the underlying molecular mechanisms by which CD44-ICD regulates prostate cancer metastasis has not been studied. There is a need for more definitive studies to understand the factors responsible for the regulation of metastasis better.

More recently, studies have identified CD44 cleavage product to be important factor regulating transcription of metastatic target genes. In breast cancer cells, sequential cleavage of CD44 resulted in nuclear accumulation of CD44-ICD [23]. Other studies in breast cancer also have shown cleavage of CD44 intracellular domain to be responsible for activation of stemness factors that promote tumorigenesis [42]. Our initial characterization in androgen receptor negative (AR^−^) PCa cells derived from bone metastasis (PC3 cells) demonstrated not only the expression of CD44 but also the formation of CD44-ICD fragments. Neither the expression of CD44 nor formation of CD44-ICD fragments were observed in AR+ cells (PCa2b and LNCaP) despite PCa2b cells are derived from bone metastasis. Androgen receptor seems to have an opposing role in the expression of CD44. To validate this statement, we used PC3 cells expressing AR. Expression of AR reduced the levels of CD44 in PC3/AR+ cells. Expression of CD44 not only increases the metastatic potential of PC3 cells [3, 24, 43, 44] but also retain stemness characteristics by regulating the expression of stem cell factors (e.g., SOX2) [7, 13, 42]. CD44 expression in PC3 cells provides metastatic, and stemness properties that regulate the tumorigenic properties and targeting CD44 will reduce or overcome metastatic and recurring PCa.

Several studies showed the association of RUNX2 with the progression of prostate and breast cancer [3, 26, 28, 45–47]. CD44 and RUNX2 expression was minimal or not observed in AR+ cells (LNCaP and PCa2b). It was shown that AR has the potential to bind RUNX2 and prevents its transcriptional function [48]. CD44 seems to have a counter-regulatory role in the activation of RUNX2 mediated events in the absence of AR in PC3 cells. As shown previously [3, 27], CD44 and RUNX2 are highly expressed in AR-negative PC3 cells. AR expression in PC3 cells reduced the levels of RUNX2, which is in line with the CD44 levels. We have shown here and previously [3] that CD44 regulates the expression of RUNX2 at mRNA and protein levels. Abrogation of CD44 cleavage by DAPT or knockdown of CD44 in PC3 cells (CD44−/−) reduced the levels of RUNX2. It is not known whether CD44 has a role in the transcriptional regulation of RUNX2, which needs further elucidation. This is the limitation of this paper.

RUNX2 has been implicated as a primary candidate to regulate adhesion and migration of cancer cells [26]. Expression of RUNX2 was observed in prostate tissue and PCa cells [26, 46, 47]. RUNX2 overexpression in breast or prostate cancer increases metastasis of these cancer cells to bone [28, 45]. It has been suggested that CD44 has been translocated into the nucleus to regulate gene transcription [18, 21]. CD44-ICD was shown to regulate the expression of several genes through its interaction with RUNX2 in breast cancer cells [18, 23]. CD44-ICD/RUNX2 interaction and their role in the regulation of transcription has not been studied in PCa cells. Therefore we proceeded to determine whether there is any interaction between CD44-ICD and RUNX2, and this interaction has any regulatory role in the expression of genes of interest in PC3 cells. We used several approaches to confirm CD44-ICD/RUNX2 interaction.

First, we determined CD44-ICD/RUNX2 interaction in the nucleus using PC3/RUNX2 cells. We showed that 20 kDa EXT-CD44 fragment was coprecipitated with RUNX2 in PC3/RUNX2 and PC3 cells but to a lesser extent, compared with 16 kDa CD44-ICD fragment. This suggests that sequential cleavage of CD44 by γ-secretase and MMPs [12, 18] is essential, and the formation of 16 kDa fragment of CD44 has more binding specificity to RUNX2. We believe that further characterization by C-terminal truncation will elucidate the sequence of CD44-ICD fragment, which has a stronger binding capacity with RUNX2 than 16 kDa CD44-ICD fragment.

Secondly, we pursued the analyses on the expression of genes as a result of CD44-ICD/RUNX2 interaction. RUNX2 has been identified as the key transcription factor for the expression of OPN and osteocalcin in osteoblasts [29, 49]. Runx2 and Ezrin expressions are closely correlative to postoperative recurrence and metastasis in patients with non-small cell lung cancer [50]. We have previously shown that CD44−/− cells displayed reduced levels of SOX2 in PC3 cells [7]. RUNX2 and MMP-9 are considered as markers of breast and prostate cancer cells, which metastasize to bone [27]. Therefore, we proceeded to determine the RNA and protein levels of SOX2, OCT4, Ezrin, MMP-9, and OPN. RUNX2 overexpression in PC3 cells increases the mRNA and protein levels of OPN and MMP-9 but had no effects on the expression levels of SOX2, OCT4, and ezrin. It is possible that CD44 signaling and not CD44-ICD/RUNX2 interaction in the nucleus may regulate the expression of these proteins. An increase in OCT4 at mRNA level of LNCaP cells suggests that the expression is androgen-dependent.

Thirdly, overexpression of RUNX2 in PC3 cells promoted migration and tumorsphere formation via upregulating genes involved in metastasis. An increase in the expression of OPN and MMP-9 suggest that RUNX2 contributes to the metastatic property of cancer cells. We also highlighted the importance of RUNX2/CD44-ICD interaction as inhibition of CD44 cleavage resulted in reduced RUNX2 expression. RUNX2 knockdown reduced the levels of MMP-9 and cell migration [27]. OPN has been implicated in the metastatic potential of various cancers. OPN-induced signaling regulates cell migration and tumor progression. This is considered as one of the novel targets for cancer therapy [51–58]. OPN expression and MMP-9 activity are linked to the progression and metastasis of prostate cancer [52, 57, 59]. OPN-overexpression in PC3 cells increased the expression of CD44 and MMP-9 [5]. Our findings in this paper provided a positive feedback loop which couples OPN expression to migration and invasion via MMP-9 [60]. OPN expression and secretion increase the levels of CD44; interaction of CD44 with MMP-9 increases the migration, colony formation, and invasion. CD44 expression regulates the expression of RUNX2, which is a critical transcriptional factor for the expression of OPN and MMP-9.

## Conclusions

Expression of CD44 and RUNX2 proteins were seen only in AR^−^ PC3 cells and not in AR+ LNCaP and PCa2b cells. CD44 regulates the expression of RUNX2. RUNX2 interaction with CD44-ICD regulates the expression of metastasis-related genes such as osteopontin and MMP-9, which had the potential to increase migration, invasion, and colony formation. Future studies will determine the nature of the interaction of CD44-ICD with RUNX2 using carboxy-terminal deletion (truncations) constructs of CD44-ICD. Identification of the CD44-ICD sequences which have a higher affinity for RUNX2 is of great importance, and that may serve as a promising therapeutic target for prostate cancer metastasis.

## Data Availability

All data generated and analyzed during this study are included in the article.

## Abbreviations

ADT: Androgen deprivation therapy
AR: Androgen receptor
CD44: Cluster of differentiation 44
CSC: Cancer stem cells
ECD: Ectodomain
EXT: External truncation
ICD: Intracellular domain
PCa: Prostate cancer
